# Probing buccal-customized epigenetic clocks to investigate socioeconomic and health disparities

**DOI:** 10.1007/s11357-025-01907-z

**Published:** 2025-10-10

**Authors:** Qiao Wu, Marta Bosanac, Maxim N. Shokhirev, Laurel Raffington

**Affiliations:** 1 Max Planck Research Group Biosocial – Biology, Social Disparities, and Development, Max Planck Institute for Human Development, Lentzeallee 94, 14195 Berlin, Germany; 2 Social and Affective Neuroscience Research Group, Laboratory of Biological Psychology, Research Unit Brain and Cognition, Faculty of Psychology and Educational Sciences, KU Leuven, B-3000 Louvain, Belgium; 3 Tally Health, New York, NY USA

**Keywords:** Biological aging, Epigenetic clock, DNA methylation, Buccal, Multimorbidity, Social determinants of health, Socioeconomic status

## Abstract

Epigenetic clocks are emerging as promising tools for examining social and health disparities. These measures are typically developed using blood DNA methylation (DNAm) data. Cheek swabs, being less invasive than blood collection, can be used to assay buccal DNAm. This study examines how buccal-originated epigenetic clocks relate to socioeconomic status (SES) and health, and compares these associations to those of blood-originated clocks applied to buccal DNAm in the German SOEP cohort (N = 1,128, aged 0–72, 57% female). We found that, unlike second- and third-generation blood-originated clocks (PCPhenoAge, PCGrimAge, and DunedinPACE), a first-generation buccal-derived clock (PedBE acceleration) and a novel second-generation buccal-derived clock trained on age, lifestyle, and health factors (CheekAge acceleration) were not significantly correlated with SES. All clocks were associated with health, and those associations were similar in magnitude across buccal- and blood-originated clocks. Our findings suggest that PedBE and CheekAge acceleration did not show stronger associations with SES and health compared to blood-originated clocks. The finding that PedBE acceleration, which is trained on chronological age in 0–20-year-olds, was associated with self-reported health and had the strongest association with multimorbidity (without cancer) is consistent with the notion that the methylome captures health-relevant processes early in ontogeny. Future studies should include multiple tissue types to further evaluate whether buccal DNAm is sensitive to socioeconomic health disparities. The development of buccal clocks may benefit from further training on aging-related biomarkers and mortality, as well as longitudinal changes in biomarkers beginning early in ontogeny.

## Background

While there is no unified definition among researchers, biological aging can be conceptualized as the progressive loss of system integrity, leading to impaired function and increased vulnerability to diseases and mortality [1, 2]. One approach to quantifying multi-system cellular biological aging involves using algorithms based on DNA methylation (DNAm), known as “epigenetic clocks”. First-generation epigenetic clocks were developed to maximize the prediction of chronological age [3]. Second-generation clocks, such as PhenoAge [4] and GrimAge [5], focus on health measures linked to morbidity and mortality. Third-generation clocks, also known as “epigenetic speedometers”, quantify the pace of aging assessed through repeated biomarker measurements of the same individuals over time (DunedinPACE-pace of aging; [6]).

Most biological age measures are developed using venous blood, which is considered the gold standard. Yet, the collection of buccal and saliva samples is gaining in popularity due to their ease of collection compared to venous blood, leading to higher participation rates, especially in hard-to-reach populations such as children [7]. Since DNAm is specific to tissue and cell types, the performance of blood-derived clocks may differ when applied to buccal samples. Blood DNAm is largely derived from leukocytes, whereas buccal samples consist primarily of epithelial cells [8–10]. In a sample of 21 individuals with both buccal and blood DNAm data, the blood-buccal correspondence of PhenoAge acceleration (cross-tissue correlation *r* = 0.25), GrimAge acceleration (*r* = 0.48), and DunedinPACE (*r* = 0.31) was found to be low-to-moderate [11].

In blood DNAm, second- and third-generation epigenetic clocks seem to outperform first-generation clocks in predicting clinical outcomes, mortality, and brain aging [12, 13]. They are also more sensitive to social determinants of health, such as demographic, socioeconomic, and behavioral characteristics [13, 14]. When applied to buccal DNAm, second- and third-generation clocks remain correlated with socioeconomic disadvantage and self-rated health, though the effect sizes appear smaller than those reported in previously published analyses of blood DNAm datasets (SES: buccal *r* = 0.08 to 0.13 versus blood *r* = 0.10 to 0.37, [11],Self-rated health: buccal *β* = 0.10 to 0.20 versus blood *β* = 0.10–0.50, [15]). This difference is presumably due to tissue variations. Therefore, it is reasonable to assume that clocks developed directly from buccal DNAm may perform better than those originating from blood when applied to buccal DNAm.

Here we evaluate whether buccal-derived epigenetic clocks are more strongly associated with SES and health compared to blood-originated clocks applied to buccal DNAm. We probe this using a first-generation buccal-derived clock, PedBE, developed in 0–20-year-olds [16] and a novel second-generation buccal-derived clock, CheekAge, which is cross-sectionally trained on age, lifestyle, and health factors [17]. This study utilizes a buccal DNAm population sample from the German SOEP-G (N = 1,128, aged 0–72, 57% female).

## Methods

The buccal DNAm subsample of SOEP-G (Genetic data in the German Socio-Economic Panel Innovation Sample) is derived from the SOEP-Core, a nationally representative, random, annual longitudinal survey of individuals residing in private households [18]. Our analytical sample included 1058 participants who contributed buccal DNAm data, selected from a total of 2,598 SOEP-G participants due to the availability of funds for DNAm data generation. The SOEP-G sample resembles the nationally representative SOEP-Core in terms of basic sociodemographic characteristics, although participants who were not born in Germany are under-represented [18]. The average age of individuals in our sample was 42.4 (*SD* = 21.2) and 57% of them were female (Table 1). The sample size for different models varied based on variable missingness and sample stratification, with final sizes detailed in each model's output.

**Table 1:** Sample Characteristics

Variable	Mean (SD) / Percentage	Sample size
Chronological age	42.4 (21.2)	1058
PedBE	30.2 (10.9)	1058
PedBE AA	-0.0 (4.4)	1058
CheekAge	59.4 (19.8)	1058
CheekAge AA	-0.0 (5.8)	1058
PC PhenoAge	99.2 (18.8)	1058
PC PhenoAge AA	-0.0 (8.6)	1058
PCGrimAge	74.3 (15.9)	1058
PCGrimAge AA	0.0 (2.5)	1058
DunedinPACE	1.6 (0.1)	1058
Age	42.4 (21.2)	1058
Female	57.5%	1045
Socioeconomic status (Z—score)	0.0 (0.8)	1045
% reporting more than 1 diseases & conditions	22.0%	797
% Dichotomous coded multimorbidity (including cancer)	7.2%	797
% Dichotomous coded multimorbidity (without cancer)	6.3%	797
Self-reported unhealthy level	2.6 (1.0)	797
% Free from functional limitation	36.6	797

Note: PC – Principal Component version; AA – Age Acceleration

The reported sample size is the number of respondents who have both DNA methylation data and the corresponding variable

### Measures

DNAm preprocessing and exclusions have been described in previous work [11]. Cell composition was estimated using HEpiDISH, which is an iterative hierarchical version of the EpiDISH R package using robust partial correlations (https://github.com/sjczheng/EpiDISH). Because epithelial cell types are the dominant cell type in buccal samples, we applied a threshold of 0.5 for epithelial cell proportions to reliably call a ‘buccal sample’ and excluded samples that failed this metric (N = 28). All samples were from the same batch. The final analytic sample size after DNAm exclusions was N = 1058.

**Epigenetic Clocks** – The clocks used in the current study include one first-generation clock, PedBE; three second-generation clocks, CheekAge, PC GrimAge, and PC PhenoAge; and one third-generation clock, DunedinPACE. Clocks that are originally developed using blood DNAm were directly computed using buccal DNAm data. Details on Clocks can be found in Table 2.

**Table 2:** Description of Epigenetic Clocks

Epigenetic Clocks	Description
PedBE	Pediatric-Buccal-Epigenetic clock (PedBE) was developed to predict chronological age in 1032 individuals aged 0-to-20 years from buccal-cell DNAm [16]. PedBE was calculated based on the published algorithm using code available at https://github.com/kobor-lab/Public-Scripts/blob/master/PedBE.Md. All 94 CpG probes were present in our dataset. PedBE acceleration was computed by residualizing PedBE for chronological age
CheekAge	CheekAge was trained on chronological age and multiple health and lifestyle factors from buccal samples of 8045 diverse adults [17]. It was further validated in internal and multiple publicly available datasets, across buccal, saliva, and blood tissues. CheekAge was computed using the CheekAge Explorer (https://cheekage.tallyhealth.com/). CheekAge acceleration was computed by residualizing CheekAge for chronological age
PC GrimAge	GrimAge was developed with a set of physiological indicators modeled from blood DNAm using machine learning analysis and then these DNA methylation algorithms along with age, sex, and a DNAm algorithm of smoking history were applied to model mortality. GrimAge represents the age in years at which average mortality risk in the Framingham Heart Study Offspring cohort matches predicted mortality risk [5] PC GrimAge was computed using DNAm principal components, which have been found to increase reliability [19], using code available at https://github.com/MorganLevineLab/PC-Clocks. PC GrimAge acceleration was computed by residualizing PC GrimAge for chronological age
PC PhenoAge	PhenoAge was first modeled from physiological markers and chronological age. This first-stage algorithm was then applied to a new sample in which it was modeled from blood DNA methylation to derive the final DNA methylation clock. PhenoAge represents the age in years at which average mortality risk in NHANES III matches the mortality risk predicted by the PhenoAge algorithm [4] PC PhenoAge was computed using DNAm principal components, which have been found to increase reliability [19], using code available at https://github.com/MorganLevineLab/PC-Clocks. PC PhenoAge acceleration was computed by residualizing PhenoAge for chronological age
DunedinPACE	DunedinPACE was developed as a DNA methylation measure of the pace of aging in the Dunedin Study birth cohort [6]. The Dunedin Study Pace of Aging is a composite phenotype derived from analysis of longitudinal change in biomarkers of organ-system integrity. Initially developed from analysis of three waves of biomarker data accumulated over a 12-year period [20]. Pace of Aging has recently been extended to a fourth measurement occasion spanning 20 years of follow-up [21]. DunedinPACE was developed from this second iteration of the Pace of Aging. Briefly, DNAm algorithm development was conducted using a subset of EPIC array probes that were also included on Illumina’s earlier 450 k array and that were identified as having relatively higher test–retest reliability [22]. Elastic-net regression machine learning analysis was used to fit Pace of Aging to DNAm data generated from blood samples collected when participants were aged 45 years. The elastic net regression produced a 173-CpG algorithm. Increments of DunedinPACE correspond to “years” of physiological change occurring per 12-months of chronological time. A value of 1 reflects the average Pace of Aging in the Dunedin Study birth cohort over the age 26–45 follow-up period. A value of 1.01 therefore reflects a pace of aging 1% faster than the Dunedin Study norm DunedinPACE was calculated based on the published algorithm using code available at https://github.com/danbelsky/DunedinPACE/. 14 of the 173 CpG probes that are part of DunedinPACE were not present in our dataset

**Socioeconomic status (SES)** – We measured SES using the average z-score of household income and education. The self-defined head of household reported monthly household net income in Euros from all sources (e.g., employment, pensions, unemployment benefits, maternity benefits, higher education grants, military or civil service pay, compulsory child support, etc.). In the 2% of cases with missing income values, information about determinants of household income and past data were used to impute estimated values. Household income was divided by the number of persons in the household and then square root transformed to correct for skewness. Given the wide age range of participants, we indexed educational attainment as the highest degree obtained by any individual in the household. Educational attainment was converted to the number of years spent in formal education.

**Health outcomes** – Multimorbidity was defined as the existence of more than one chronic disease/health condition. Three multimorbidity measures were used in the current study: (1) Multimorbidity (without cancer) included the self-reported doctor’s diagnosis of diabetes, cardiopathy, stroke, and high blood pressure; (2) Multimorbidity (with cancer) additionally included the diagnosis of cancer; (3) Self-reported diseases & conditions further included sleep disturbance, migraine, asthma, depressive disorder, dementia, joint disorder, chronic back pain, and other illnesses. These multimorbidity measures were dichotomously coded (Having no or only one item = 0; Having more than one item = 1). The self-reported unhealthy level is a score based on the survey question where respondents were asked to describe their state of health, with a higher score indicating a worse health state (1 = very good, 2 = good, 3 = satisfactory, 4 = poor, 5 = bad). Limitation of daily activities is a dichotomous outcome based on the question where respondents were asked how severely their health restricts them from ascending stairs, makes tasks tiring, and limits their normal daily activities (1 = yes strongly limited, 2 = yes somewhat limited, 3 = no, not limited at all). It was coded as 1 when the respondent’s normal daily activities are strongly or somewhat limited and coded as 0 when not limited at all. Health outcomes are only available for adult respondents (age > 18).

### Statistical Analysis

Our statistical analyses proceeded in three steps. *First*, we examined associations between epigenetic age indicated by the clocks (not residualized for chronological age) and chronological age to ensure that data in our sample followed the expected patterns of associations found in prior studies. We assessed the associations regressing each clock on chronological age separately.

*Second*, we explored the degree to which different epigenetic age accelerations (residualized for chronological age) and the pace of aging were correlated with each other.

*Third*, we examined whether epigenetic age acceleration was associated with SES (average z-score of household income and education), adjusting for age and sex as covariates in Ordinary Least Squares (OLS) regression models. Due to the wide age range of the sample, and since epigenetic clocks were developed in samples with different age ranges, we further tested whether the association between SES and clocks differed by chronological age by adding an interaction term between clocks and continuous chronological age to the OLS regression models. To illustrate significant interactions, we stratified our sample by applying an age 45 years grouping, which is the common threshold used to separate children/young adults and middle-aged/older adults.

*Fourth*, we probed whether epigenetic age acceleration was associated with health among adult respondents, adjusted for age and sex as covariates. We were only able to test the associations with health among adult because health data for children was unavailable. OLS regression models were employed for the continuous outcome (self-reported unhealthy level) and standardized coefficients were reported for comparison. Logistic regression models were used for dichotomous outcomes (multimorbidity measures and limitation of daily activities), where epigenetic clocks were z-score standardized for comparison, and odds ratios were reported. Standardizing the clocks ensures comparability of effect sizes across models and accounts for differences in clock variance, as recommended for robust interpretation of regression-based analyses in studies of biological aging (Teschendorff & Horvath, 2025).

Given the wide age range of the sample, and since epigenetic clocks were developed in samples with different age ranges, we further tested whether the association between clocks and health differed by chronological age by adding an interaction term between clocks and continuous chronological age to the OLS regression models. To illustrate significant interactions, we stratified our sample by applying an age 45 years grouping.

All analyses were conducted in R version 4.4.2.

## Results

1. Associations between epigenetic clocks and chronological age

**Fig. 1:**
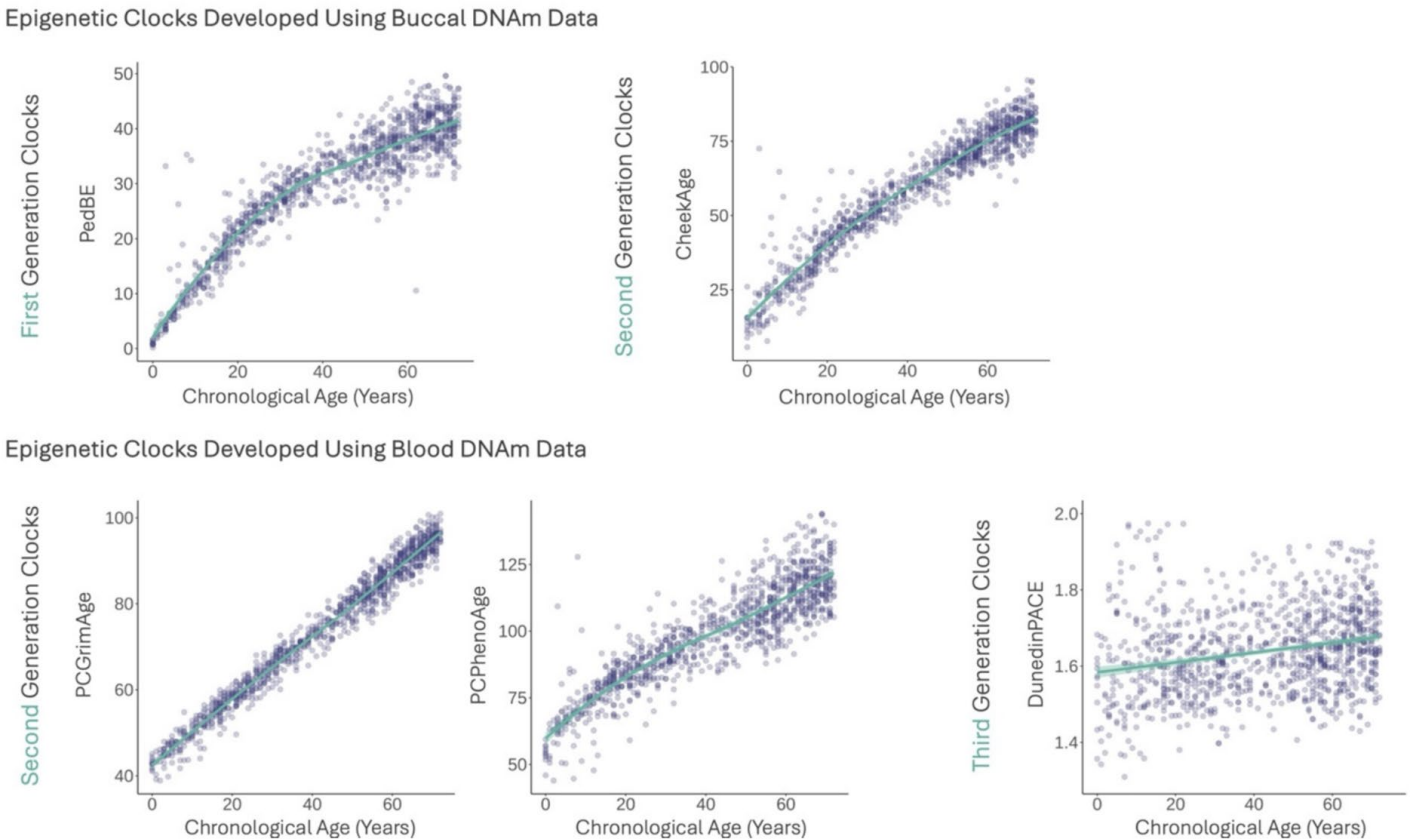
Scatterplots depicting the association between Epigenetic Clocks developed using buccal DNAm (top panel) or blood DNAm (bottom panel and Chronological Age. *Note. Pearson correlation coefficients and p values were as follows: PedBE: r* = *0.91, p* < *0.001; CheekAge: r* = *0.96, p* < *0.001; PCPhenoAge: r* = *0.89, p* < *0.001; PCGrimAge: r* = *0.99, p* < *0.001; DunedinPACE: r* = *0.24, p* < *0.001*

We first examined epigenetic age associations with chronological age. Figure 1 shows that PedBE (*r* = 0.91, *p* < 0.001) and CheekAge (*r* = 0.96, *p* < 0.001) were very highly correlated with chronological age, similar to PCGrimAge (*r* = 0.99, *p* < 0.001) and PCPhenoAge (*r* = 0.89, *p* < 0.001). In contrast, DunedinPACE exhibited a modest correlation with chronological age (*r* = 0.24, *p* < 0.001). This is expected, as DunedinPACE estimates the pace of aging within individuals, which does not necessarily correlate strongly with chronological age. Instead, it indicates that the pace of aging tends to increase with advancing age.

(2) Associations between epigenetic age acceleration and pace of aging clocks

**Fig. 2:**
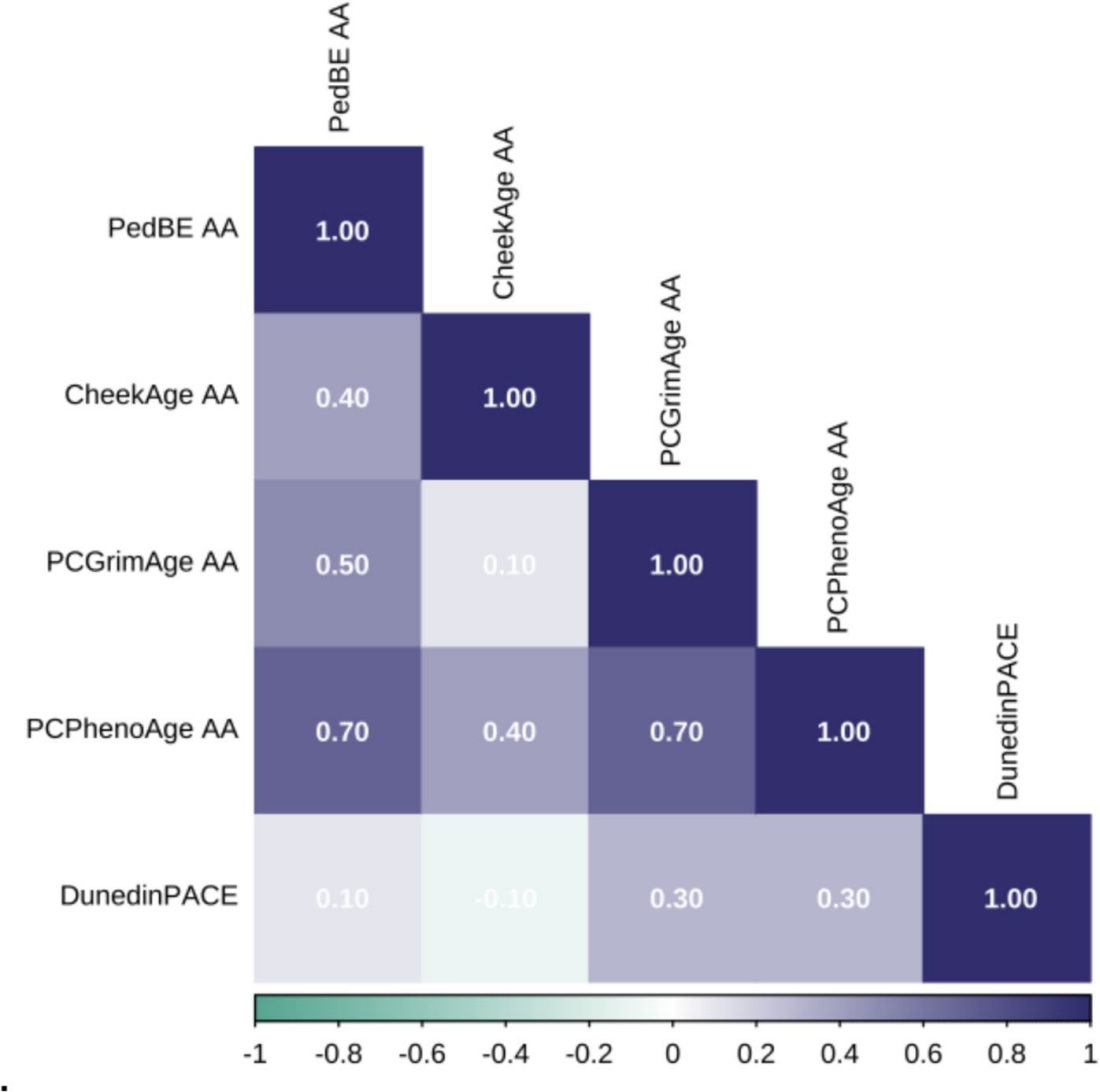
Correlation Matrix of different epigenetic clock measures of biological age acceleration and pace of aging. *Note*. *PC – Principal Component version; AA – Age Acceleration Pearson correlation coefficients are shown.*

Next, we explored the degree to which different epigenetic clock measures of age acceleration (residualized for chronological age) and pace of aging were correlated with each other. Figure 2 indicates that PedBE AA was more strongly correlated with blood-originated clocks (*r*’s range from 0.50 to 0.70) than CheekAge AA (*r*’s range from 0.10 to 0.40). PedBE AA and CheekAge AA were moderately correlated with each other (*r* = 0.40). Buccal versions of PCPhenoAge AA and GrimAge AA were strongly correlated with each other (*r* = 0.7). Buccal DunedinPACE stood out as being uncorrelated or only weakly correlated with all other clocks (*r*’s range from -0.10 to 0.30).

(3) Associations between epigenetic clocks and socioeconomic status

**Fig. 3:**
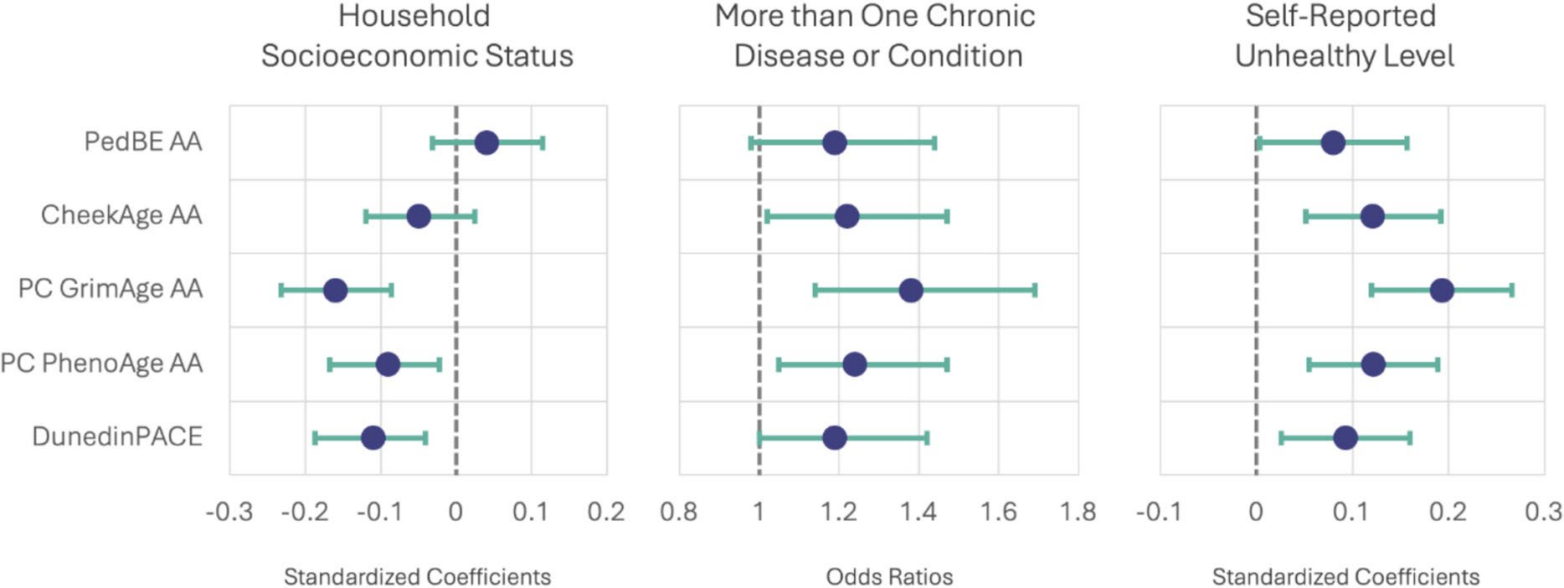
Associations of Epigenetic Clocks with Socioeconomic Status and Health. *Note: PC – Principal Component version; AA – Age Acceleration. In the first panel, household socioeconomic status (SES) was used as the independent variable, and epigenetic clocks were used as dependent variables, separately in each model, adjusted for age and sex. The sample includes respondents of all ages. Household SES was a continuous variable so standardized coefficients were shown. In the second panel, the self-reported unhealthy level was used as the dependent variable, and epigenetic clocks were used as independent variables, separately in each model, adjusted for age and sex. The sample includes adult respondents. The self-reported unhealthy level was a continuous variable so standardized coefficients were shown. In the third panel, having more than one chronic disease or condition was used as the dependent variable, and epigenetic clocks were used as independent variables, separately in each model, adjusted for age and sex. The sample includes adult respondents. Having more than one chronic disease or condition was a dichotomous variable so odds ratios were shown. Clocks were z-score standardized for comparison*

We examined if SES was associated with CheekAge AA and PedBE AA in a manner similar to blood-originated clocks applied to buccal DNAm. We found that SES was not significantly associated with either CheekAge AA or PedBE AA. In contrast, blood-originated clocks indicated that lower SES was linked to accelerated epigenetic aging and a faster pace of aging (*β*s range from -0.18 to -0.10, all *p*-values ≤ 0.009). These associations between SES and each clock are detailed in Table 3 (section A) and illustrated in Fig. 3.

**Table 3:** Associations of Epigenetic Clocks with SES (Average of Z-Scored Household Income and Education)

Epigenetic Clocks	Standardized Coefficient	P Value	CI Lower	CI Upper
A. All Ages, N = 1045				
PedBE AA	0.04	0.273	-0.032	0.115
CheekAge AA	-0.05	0.192	-0.122	0.025
PCGrimAge AA	**-0.18**	**0.000**	-0.243	-0.110
PCPhenoAge AA	**-0.10**	**0.009**	-0.171	-0.024
DunedinPACE	**-0.11**	**0.002**	-0.187	-0.040
B. Age < = 45 years, N = 504				
PedBE AA	0.05	0.273	-0.042	0.149
CheekAge AA	-0.07	0.292	-0.187	0.056
PCGrimAge AA	-0.06	0.197	-0.162	0.033
PCPhenoAge AA	-0.02	0.654	-0.133	0.084
DunedinPACE	N/A	N/A	N/A	N/A
C. Age > 45 years, N = 541				
PedBE AA	**-0.10**	**0.021**	-0.187	-0.015
CheekAge AA	**-0.13**	**0.002**	-0.209	-0.045
PCGrimAge AA	**-0.26**	**0.000**	-0.349	-0.163
PCPhenoAge AA	**-0.18**	**0.000**	-0.283	-0.082
DunedinPACE	N/A	N/A	N/A	N/A

Note: PC – Principal Component version; AA – Age Acceleration; CI – Confidence Interval

Each line corresponds to a separate model

All models were adjusted for age and sexFor DunedinPACE, the age interaction was not statistically significant, so no stratified models were included

We then probed if associations between SES and CheekAge AA or PedBe AA differed by age, which we had previously found using blood-originated clocks in this cohort [11]. The interaction terms between SES and chronological age suggested statistically significant but small age differences in the association between SES and clocks except for DunedinPACE (*β*s range from -0.01 to 0.00, all *p*-values ≤ 0.023).

To further illustrate these interactions, we stratified our analytic sample by age 45, separating children and young adults from middle-aged and older adults for all clocks except for DunedinPACE (Table 2**,** sections B & C). Among participants at or younger than 45 years (children and young adults, N = 504), SES was not significantly associated with CheekAge AA, PedBe AA, PCGrimAge AA, or PCPhenoAge AA. Among participants older than 45 (middle-aged and older adults, N = 541), SES was significantly associated with both CheekAge AA (*β* = -0.13, *p* = 0.002) and PedBE AA (*β* = -0.10, *p* = 0.021). The magnitude of these associations was similar to PCGrimAge AA (*β* = -0.26, *p* < 0.001) and PCPhenoAge AA (*β* = -0.18, *p* < 0.001).

(4) Associations between epigenetic clocks and health outcomes

We examined the associations between CheekAge AA and PedBe AA with health outcomes available for adult respondents (age > 18) and compared these associations to those of blood-derived epigenetic clocks applied to buccal DNAm (Table 4**,** section A). Figure 1 shows that CheekAge AA was significantly associated with higher odds of self-reported diseases and conditions (*OR* = 1.22, *p* = 0.029) and an increased self-reported unhealthy level (*β* = 0.12, *p* = 0.001). Similarly, PedBE AA was significantly associated with multimorbidity excluding cancer (*OR* = 1.46, *p* = 0.026) and a higher self-reported unhealthy level (*β* = 0.08, *p* = 0.042).

**Table 4:** Associations of Epigenetic Clocks with Health Outcomes

Epigenetic Clocks	Self-Reported Diseases & Conditions	Multimorbidity (Including Cancer)	Multimorbidity (without Cancer)	Limitation of Daily Activities	Self-Reported Unhealthy Level
	OR (p value)	OR (p value)	OR (p value)	OR (p value)	Std. Coef. (p value)
A. Age > 18 years, N = 797					
PedBE AA	1.19 (0.089)	1.31 (0.083)	**1.46 (0.026)**	1.03 (0.776)	**0.08 (0.042)**
CheekAge AA	**1.22 (0.029)**	1.26 (0.112)	1.28 (0.112)	1.16 (0.070)	**0.12 (0.001)**
PCGrimAge AA	**1.38 (0.001)**	1.25 (0.155)	1.23 (0.200)	**1.23 (0.016)**	**0.19 (0.000)**
PCPhenoAge AA	**1.24 (0.011)**	1.20 (0.154)	1.30 (0.054)	1.14 (0.099)	**0.12 (0.000)**
DunedinPACE	**1.20 (0.049)**	**1.46 (0.010)**	**1.39 (0.033)**	1.09 (0.299)	**0.09 (0.007)**
B. Age < = 45 years, N = 276					
PedBE AA	N/A	N/A	N/A	N/A	-0.03 (0.730)
CheekAge AA	N/A	N/A	N/A	N/A	-0.01 (0.908)
PCGrimAge AA	N/A	N/A	N/A	N/A	N/A
PCPhenoAge AA	N/A	N/A	N/A	N/A	N/A
DunedinPACE	N/A	N/A	N/A	N/A	N/A
C. Age > 45 years, N = 521					
PedBE AA	N/A	N/A	N/A	N/A	**0.10 (0.044)**
CheekAge AA	N/A	N/A	N/A	N/A	**0.16 (0.001)**
PCGrimAge AA	N/A	N/A	N/A	N/A	N/A
PCPhenoAge AA	N/A	N/A	N/A	N/A	N/A
DunedinPACE	N/A	N/A	N/A	N/A	N/A

*Note: PC – Principal Component version; AA – Age Acceleration; OR – Odds Ratio; Std. Coef. – Standardized Coefficient. P values are reported in parentheses. For continuous outcomes, standardized coefficients are reported. For dichotomous outcomes, odds ratios are reported, and epigenetic clocks are standardized for comparison. Each cell corresponds to a separate model. All models were adjusted for age and sex. Age interactions were only significant for PedBE AA and CheekAge AA, age-stratified models were only included for those two clocks*

All blood-originated epigenetic clocks were significantly associated with the self-reported unhealthy level, and the effect sizes of CheekAge AA and PedBE AA were similar compared to those of blood-originated clocks (*β*s of blood-originated clocks range from 0.09 to 0.19, all *p*-values ≤ 0.007). All blood-originated clocks were also significantly associated with having self-reported diseases and conditions, and the effect sizes of CheekAge AA were similar compared to those of blood-originated clocks (*OR*s of blood-originated clocks range from 1.20 to 1.38, all *p*-values ≤ 0.049). In addition to PedBE AA, only DunedinPACE (*OR* = 1.39, p = 0.033) was also significantly associated with multimorbidity excluding cancer, and the effect size of PedBE AA was relatively larger. Only DunedinPACE was significantly associated with multimorbidity including cancer (*OR* = 1.46, *p* = 0.010). Only PC GrimAge AA was significantly associated with the limitation of daily activities (*OR* = 1.23, *p* = 0.016).

We further tested if associations between buccal-derived epigenetic clocks and health outcomes differed by age. We only found statistically significant interactions between PedBE AA and chronological age (*β* = 0.01, *p* = 0.026) and between CheekAge AA and chronological age (*β* = 0.01, *p* = 0.003) on self-reported unhealthy level. We further examined these significant interactions by stratifying our sample by age 45, separating the sample into children & young adults and middle-aged & older adults for PedBE AA and CheekAge AA (Table 4 sections B & C). For young adults, neither of the two clocks was significantly associated with any health outcomes. Among middle-aged and older adults, the effect sizes of both PedBE AA (*β* = 0.10, *p* = 0.044) and CheekAge AA (*β* = 0.16, *p* = 0.001) with the self-reported unhealthy level became larger compared to those among all adults.

## Discussion

We examined how buccal-originated epigenetic clocks related to socioeconomic status (SES) and to health, and compared these associations to those of blood-originated clocks applied to buccal DNA methylation in the German SOEP-G cohort. We found that, unlike blood-originated second- and third-generation clocks (PCPhenoAge acceleration, PCGrimAge acceleration, DunedinPACE), neither the first-generation (PedBE acceleration) nor the second-generation (CheekAge acceleration) buccal-derived clocks showed significant correlations with SES. All biological aging measures, except for DunedinPACE, demonstrated stronger associations with SES among middle-aged and older adults compared to children and young adults.

Moreover, our results indicated that all clocks were correlated with health outcomes. The associations of PedBE and CheekAge accelerations with self-reported unhealthy levels were more pronounced among middle-aged and older adults than among young adults. Notably, PedBE acceleration, which was developed in children, had a slightly stronger association with multimorbidity (*OR* = 1.46) compared to other clocks (*OR*s range from 1.23 to 1.39).

Overall, our findings suggest that the currently available epigenetic clocks developed in buccal DNAm have associations with SES and health outcomes similar in magnitude to those of blood-originated clocks when applied to buccal DNAm. This is somewhat surprising, given that buccal-derived clocks are optimized for tissue specificity, while blood-originated clocks exhibit only low-to-moderate cross-tissue correspondence when applied to buccal DNAm [11].

There are several potential explanations for these results. First, when the primary goal is to measure biological aging, buccal-customized algorithms may require further optimization. This optimization should be similar to the top-performing blood-derived “second and third generation” algorithms, which have shown strong associations with both SES and aging-specific outcomes [12, 13, 23–25]. PedBE is a first-generation clock designed solely to predict chronological age. In contrast, CheekAge is a second-generation clock trained that balances chronological age prediction with sensitivity to a wide range of lifestyle and health variables. This multi-variate approach may account for its superior performance compared to several first-generation clocks and its comparable performance to PhenoAge in predicting various health factors from blood and other tissue datasets [26].

However, the health information used to develop CheekAge, such as self-rated health, self-perceived aging, stress level, and frequency of illness, was broad and not specific to biological aging. While CheekAge captures a wide range of health signals, its ability to measure biological aging may be compromised, leading to limited sensitivity to other factors contributing to gradual, chronic, and cumulative health decline, including factors associated with socioeconomic disadvantage. This notion is supported by a recent meta-analysis showing stronger SES associations with GrimAge acceleration and DunedinPACE compared to other clocks [25]. To enhance the prediction of biological aging, future buccal-derived epigenetic clocks could benefit from associating DNAm with endpoint aging outcomes, similar to GrimAge, and/or longitudinal changes in aging biomarkers, akin to DunedinPACE.

Somewhat surprisingly, PedBE acceleration, which quantifies methylation differences across various life stages from infancy to emerging adulthood, was associated with health in older adults. It showed the strongest association with a multimorbidity measure that captures the coexistence of conditions like diabetes, cardiopathy, stroke, and high blood pressure, excluding cancer. In infants, accelerated PedBE has been associated with gestational age and birthweight [16]. This study further suggests that the epigenetic mechanisms underlying early-life development, which are associated with gestational age and birthweight, may have a stronger connection to multimorbidity than some clock algorithms designed to capture biological aging in adults.

While researchers differ on when biological aging begins, many propose it starts near the onset of development, theorizing that aging and development may run parallel or even overlap biologically [27]. Our findings align with this view, indicating that the methylome captures early ontogenetic processes related to later-life cardiometabolic health [7, 28, 29]. These insights suggest that future development of aging-related biomarkers could benefit from including information on childhood or biosamples of children.

A second potential explanation for our results is the difference in sensitivity to aging processes between blood and buccal samples. Blood samples are predominantly composed of leukocytes, whereas buccal samples contain a mixture of leukocytes and epithelial cells. Although both sample types share the same dominant leukocytes, such as lymphocytes and neutrophils, buccal samples have a much lower proportion of leukocytes, with epithelial cells making up over 80% of the cellular content [9][8–10].

The association of blood-originated epigenetic clocks with SES and health, despite cross-tissue signal loss, suggests that methylation patterns in leukocytes could be crucial for quantifying multi-system biological aging. Although the correspondence between blood and buccal samples for blood-originated clocks is low to moderate [11], these clocks show stronger cross-tissue correspondence when applied to saliva [30], which contains a higher proportion of leukocytes compared to buccal samples [9, 10, 31]. Therefore, using saliva samples for DNAm extraction could be a better non-invasive alternative to retain epigenetic signals from leukocytes when blood collection is not feasible. Blood circulates throughout the body, capturing signals from multiple organs and tissues, thus providing a comprehensive reflection of multi-system organ integrity.

Buccal samples, despite not being ideal for measuring certain aging aspects due to their relatively low leukocyte content, remain a highly accessible tissue type that may be sensitive to specific exposures and biological mechanisms. Unlike blood, the immune and epithelial cells in buccal samples may be more directly exposed to certain environmental factors such as smoking and diet, potentially capturing unique epigenetic signatures. Given the shared ectodermal origin of epithelial and brain cells, buccal DNAm biomarkers could potentially provide insights into early neural development [32]. However, the few studies evaluating genome-wide cross-tissue correlations have not fully supported this hypothesized potential: Buccal-brain correlations did not outperform blood–brain or saliva-brain correlations [33, 34]. Nonetheless, buccal samples may hold value beyond prediction precision alone,in some studies, including specific populations might be prioritized over maximizing prediction accuracy.

We acknowledge several limitations of our study. First, our comparison of blood- and buccal-derived biological age measures was restricted to buccal samples. Future studies should compare the associations of epigenetic clocks with SES and health outcomes using paired buccal and blood samples from the same individuals. Second, the SOEP survey, designed to include participants across all age groups, does not intend to include a comprehensive collection of health measures typically utilized in aging research and lacks specific aging biomarkers. In addition, all the health measures used in the current study rely on self-reports. Although self-reports are commonly used and generally considered valid in population surveys [35, 36], future studies could leverage health records for more objective health and morbidity information. Lastly, our analyses relied on cross-sectional data, which precludes any inference about the causal directions of the estimated associations.

In conclusion, our study found that buccal-originated epigenetic clocks did not exhibit stronger associations with SES and health compared to blood-originated clocks when applied to buccal DNAm. Similar to the advancements seen in second- and third-generation blood biomarkers of aging, the development of buccal clocks could benefit from being trained on aging-specific biomarkers, mortality data, and longitudinal biomarker changes. Furthermore, studies that include paired tissues from the same individuals should critically assess whether buccal clocks are inherently less sensitive to multi-system aging processes and socioeconomic health disparities compared to blood clocks.

## Data Availability

The current study performed secondary data analysis using the SOEP-G data. Interested data users should contact user support (https://www.diw.de/en/diw_01.c.601584.en/data_access.html).
